# Exploring the Relationship Between the Indices of Body Composition With Grip Strength Performance and Peak VO2

**DOI:** 10.7759/cureus.40874

**Published:** 2023-06-23

**Authors:** Sushmitha S, Ruchi Kothari, Gaurav Mittal, Maitri Gopani, Prashanth A, Pradeep Bokariya, Sai Shanmukh Vemparala, Shubhi Tamrakar, Abishek S, Bennita A

**Affiliations:** 1 Anatomy, Mahatma Gandhi Institute of Medical Sciences, Wardha, IND; 2 Physiology, Mahatma Gandhi Institute of Medical Sciences, Wardha, IND; 3 Physiology, Andhra Medical College, Visakhapatnam, IND; 4 Physiology, Dr. DY Patil School of Medicine, Mumbai, IND; 5 Physical Medicine and Rehabilitation, SRM Medical College Hospital & Research Centre, Kattankulathur, IND

**Keywords:** a body shape index, body surface area, body mass index: bmi, waist hip ratio, hand grip strength, peak vo2

## Abstract

Background

The importance of measurements of body composition in terms of various indices including Body Mass Index (BMI), Body Surface Area (BSA), Body Size Index (BSI), and Waist to Height ratio (WtHR) in the diagnosis of health risks and mortality outcome analysis has largely been limited to their use relating to determining abdominal obesity. The understanding of the extent of implications of the newer, underutilized indices of body composition is deficient. Peak VO_2 _(maximal oxygen uptake) majorly serves for the evaluation of the measure of aerobic capacity. Grip strength performance is a simple, primary, objective predictor of overall physical status and muscular and cardiovascular fitness.

This study aimed to derive the relationship between a gamut of parameters such as BMI, BSA, WtHR, BSI, grip strength performance and peak VO_2_ investigated using the latest scientific methodology in a cross-section of the population in a rural tertiary care center.

Methodology

This study was a descriptive, cross-sectional study carried out in a rural medical college in central India. Sixty participants from the healthcare setting were considered eligible for the study within the age group of 18 to 45 years. Anthropometric assessments like height (in cm), weight (in kg), waist circumference (in cm), and BMI were carried out. BSA, WtHR, and BSI were calculated using the respective formulae. VO_2_ max (maximal oxygen uptake) recordings were done using the treadmill/ergometer and metabolic module of LabChart software (Bella Vista, New South Wales, Australia). Grip Strength Performance was quantified by measuring the amount of static force with which the hand is able to squeeze a transducer. It was measured using Grip Force Transducer (MLT004 / ST) from AD Instruments (Bella Vista, New South Wales, Australia).

Results

Upon analysis, a signiﬁcant negative correlation was obtained between BSI and BMI (r= -0.51, p<0.0001) whereas a significant positive correlation was found between BSA and BMI (r= 0.71, p< 0.0001). A significant correlation was also seen between WtHR and BMI (r= 0.71, p< 0.0001) while a negative significant correlation between peak VO_2_ and BMI (r= -024,p=0.0425) was deduced. Similarly, a negative correlation was evident between BSA and BMI (r= -0.46, p=0.0002) with a positive correlation between WtHR and BSA (r= 0.30,p=0.0188). Grip strength performance positively correlated with BSA (r= 0.58, p< 0.0001) whereas peak VO_2_ showed a significant negative correlation with WtHR (r= -026,p=0.043). There was also a positive significant correlation between grip strength performance and peak VO_2_ (r= 0.37, p=0.0033)

Conclusion

The study determined the relationships of grip strength performance and peak VO_2_, with the body composition indices in order to provide an overview of the mortal risks of an individual which might mediate the prognosis. Based on the relative independence of BSI with peak VO_2_ and grip strength performance, the unification of these parameters can help assess the overall health of an individual.

## Introduction

Indicators of health that can be determined conveniently and at low cost continue to play a central role in the epidemiological and clinical assessment of the chronic and degenerative conditions that cause extensive morbidity and mortality [1]. The currently unexplored indicators of disease are grip strength performance, maximal aerobic capacity (peak VO_2_), and indices of body composition. The realm of the different anthropometric parameters of body composition especially Body Mass Index (BMI), Body Surface Area (BSA), Body Size Index (BSI), Waist Circumference (WC), and Waist to Height ratio (WtHR) has largely been restricted to the measurement of abdominal obesity and estimating the risk of development of morbidities. A Body Shape Index (ABSI) is an anthropometric parameter calculated by dividing waist circumference (WC) by its estimate obtained from allometric regression of weight and height. BSA is the measured or calculated surface area of a human body which is an indicator of metabolic mass than body weight and is least affected by abnormal adipose tissue. BMI is a measure of body fat. WC is a risk predictor stemming from abdominal obesity. The waist-to-hip ratio is another way of assessing abdominal obesity, and this measure correlates with cardiovascular risk [2,3]. 

Understanding the extent of implications of the newer, underutilized indices of body composition is deficient. Moreover, the Centre for Disease Control and Prevention states that though BMI is a reasonable indicator of body fat for adults, it should primarily only be employed to track the weight status of an individual and should be used as a screening tool to identify weight-related problems in adults. With a greater understanding of the shortcomings of BMI as a screening tool for obesity, newer indices of body composition, such as WtHR, BSA, BSI must be considered for application in the clinical setting [4].

Grip strength performance is a direct and efficient measure of skeletal muscle status [5]. It provides valuable information on the function and status of the musculoskeletal system. However, the measurement of grip strength performance also reveals crucial information about the overall health of the subject [5]. Poor grip strength performance has been linked to mortality [6] and morbidity, sarcopenia, and cachexia [7]. While the focus of a large number of research based on grip strength was on quantifying the mortality risk in older adults [8] (beyond the age of 45 years), a few studies have also established the higher risk of poor health (mortality and morbidity) with poor grip strength measured at adolescence and middle age [9,10].

Recently Celis-Morales et.al. established the value of grip strength performance analysis in their UK Biobank study, which firmly confirmed, on the basis of the analysis of a cohort of half a million individuals, that grip strength is strongly and inversely associated with all-cause mortality and incidence of and mortality from cardiovascular disease, respiratory disease, chronic obstructive pulmonary disease, all cancers and its subtypes, including colorectal, lung, and breast cancer, with associations being modestly stronger in the younger age groups. They also found that the additional analysis of grip strength performance improves the prediction ability for all causes of morbidity and mortality of cardiovascular health diseases and suggest its use alternatively in setups where biochemical screening might not be an option [11].

Peak oxygen uptake is a function of oxygen delivery and utilization [12]. Peak VO_2_ is known to be a gold standard in the measure of exercise capacity and life expectancy [7,13], through its domain of occupational and sports therapy. It indicates an individual’s ability to perform a physical task via the estimation of the maximum oxygen uptake during a graded exercise [14]. These implications make peak VO_2_ a powerful tool for the measurement of aerobic capacity, even in the general populace, especially with recent studies linking lower peak VO_2_ with the risk of developing cardiovascular disease [13,15].

But while peak VO_2_ is a critical prognostic factor in patients with cardiovascular disease [13], it is unclear how well it predicts mortality in healthy persons. There is also uncertainty regarding the predictive power of aerobic capacity relative to other clinical variables. Based on the trends of peak VO_2_ in disease conditions like diabetes mellitus, neoplasia, and hypertension [13,15], when physical fitness is checked, the functional status of all systems like the cardiovascular, respiratory, neuroendocrine, muscular, and circulatory systems is actually being assessed [16].

Evidently, in isolation, each of these parameters is known to be a good indicator of a person's overall physical condition and is associated with similar health outcomes. yet their clinical application has not been explored. Moreover, the understanding of the extent of implications of the newer, underutilized indices of body composition is deficient [17]. Hence, we identified the need to probe the relationship between these metrics and determine the scope for the development of a composite analytical tool.

This study attempts to derive a relationship between these parameters and establish a deterministic variable. The novelty of this study lies in its measurement of body composition, by including a gamut of parameters such as BSA, BSI, and WtHR, since evaluation using a single parameter like BMI may lead to misdiagnosis of a healthy adult as an obese person [18], and using the latest scientific methodology to calculate grip strength performance and peak VO_2_ of the subjects.

The objective of this study was to explore the nature of the relationship between the different indices of body composition, muscular fitness (using grip strength performance), and maximum aerobic capacity (using peak VO_2_) in a cross-section of the population of a rural tertiary care center in central India, and to exploit the understanding deduced thereby, for a clinical setting, by establishing a potential diagnostic/ prognostic tool.

## Materials and methods

Study design and population selection

This was an observational, cross-sectional study. The Strengthening the Reporting of Observational Studies in Epidemiology (STROBE) guidelines for the cross-sectional study were used for reporting and preparing the manuscript. The study was carried out in the Department of Anatomy in collaboration with the Department of Physiology of a rural medical college in central India. A total of sixty participants within the age group of 18-45 years from the healthcare setting were recruited in the study using the convenient sampling technique. Subjects who were included in the study were those who gave written informed consent while the subjects suffering from diseases such as respiratory illnesses (asthma, emphysema, chronic obstructive pulmonary disease), chronic hypertension, diabetes, musculoskeletal diseases, cardiovascular diseases, diagnosed psychiatric disorders, subjects with musculoskeletal pain on test day, and those who refused to give consent were excluded. The study commenced only after review and approval by the Institutional Ethics Committee. A signed written informed consent from all study participants prior to their testing was ensured.

Data sources and measurement of variables

Anthropometric Variables

The age of the subject was noted in years. The standing height of the subjects was recorded in centimeters (cm) while they were barefoot, with their heels together. Weight in kilograms (kg) was measured in a standing position with light clothes and without footwear. Waist circumference (WC) was measured using a measuring tape over bare skin or light undergarments.

Measurement sites were obtained with the subject assuming a standing position. Waist circumference was measured halfway between the lower border of the ribs and the iliac crest in the horizontal plane. Two measurements to the nearest 0.5 cm were recorded. If the variation between the measurements were >2cm, a third measurement was taken. The mean of the two closest measurements was calculated. Body mass index (BMI) was recorded by Phoenix Height Weight Body Mass Index Machine (Model: PBMI -200).

BSI has been derived from waist circumference and is said to be a better index. Body Shape Index (BSI) was calculated using the formula [19] - 

$BSI = WC/ [(BMI)^{2/3} X (Height)^{1/2}]$

Body Surface Area (BSA) was calculated using the formula [19]-

$BSA = 0.20247 X Height ^{0.725} X Weight ^{0.425}$

Waist-to-height ratio (WtHR): was calculated by dividing WC by height using the formula - 

$WtHR=WC/Height$

WtHR has only recently gained attention as an anthropometric index for measuring central adiposity. WtHR is a more sensitive, cheap, easy-to-use, universal screening tool that is a less age-dependent index to identify individuals with increased cardiometabolic risk [18].

Peak VO₂

Power Lab 8/35 System from AD Instruments, Australia was employed for data acquisition of respiratory gas concentrations and airflow during the use of an ergometer by the subjects. The Metabolic Module for LabChart Software was used primarily for determining the peak VO₂. It recorded the inspired or expired airflow from a pneumotach and CO_2_ and O_2_ concentrations from the gas analyzer by the expired air in the gas mixing chamber.

The subjects completed a 5-minute warm-up at 25 watts prior to maximal testing on a motorized bicycle ergometer (Aerofit AF 176U). Then an incremental cycle ergometer test was conducted to measure peak VO₂. The pedal rate was maintained at 60 rpm throughout the testing. The peak test began at unloaded cycling, 60 cycles per minute for 2 minutes. Thereafter, the workload was increased by 29 W (0.5 kg) every 2 minutes until 118 W. From this point on, power output was increased by 15 W (0.25 kg) until volitional termination or a drop in pedal rate of 5 cycles per minute was found. 

Grip Strength Performance

Grip Strength Performance is quantified by measuring the amount of static force with which the hand is able to squeeze a transducer. It was measured using a grip force transducer (MLT004/ST) from AD Instruments, Australia. A single value of maximum voluntary contraction, which represents 100% of the handgrip strength, was noted. The mean value of handgrip strength was calculated from a set of values obtained during a period of 0 to 2 minutes with an interval of 20 sec each. Blood pressure (BP) and heart rate (HR) were recorded before and after the exercise. The raw data obtained by the Power lab data recording system was analyzed by Lab Chart software and was expressed in Newton (N). The subject was briefed about the entire procedure to alleviate any apprehension. Relevant medical history was recorded by the baseline assessment questionnaire. Anthropometric parameters and basic vitals (BP, HR, and respiratory rate) were recorded, followed by the measurement of peak VO₂ and grip strength performance by the aforementioned method.

Statistical analysis

The data obtained were entered into a Microsoft Excel spreadsheet and then we computed the mean and standard deviation. The data were analyzed by using the SPSS (Statistical Package for Social Sciences) Version 20.0, SPSS Inc, Chicago, Illinois, USA. The study parameters were correlated using Pearson correlation. The data was normally distributed. The correlation coefficient value “r” either positive (direct correlation) or negative (inverse correlation) was calculated between two quantitative variables with its t-test for testing its significance. The level of significance was kept as p<0.05.

## Results

The study included 60 individuals between the ages of 18 and 45 years who were apparently healthy on test day and satisfied the selection criteria mentioned above. The mean age of the participants was 26.80 with a standard deviation of 6.68 years. The descriptive statistics of the subjects under study are enumerated in Table 1. The mean and standard deviation of variables namely the hand grip strength of the subjects was 92.06 ± 24.95 and Peak VO₂ was 39.30 ± 5.63 ml/min/kg.

**Table 1:** Demographic parameters of the study population BMI - Body Mass Index BSA - Body Surface Area BSI - Body Size Index WtHR - Waist to Height ratio

S.No.	Parameter	Average	Std Dev
1.	Height (cm)	170.22	6.64
2.	Weight (kg)	64.13	10.48
3.	Waist circumference (cm)	84.78	7.45
4.	Peak VO₂ (ml/min/kg)	39.30	5.63
5.	Peak HR (bpm)	162.51	14.03
6.	Mean grip strength (N)	92.06	24.95
7.	BMI (kg/m^2^)	22.13	3.37
8.	BSA (m^2^)	1.74	0.15
9.	BSI	0.08	0.01
10.	WtHR	0.50	0.05

Table 2 shows the correlation analyses between the different parameters studied. The indices of body composition correlated well with each other. The correlation of BSA and WtHR with BMI was a highly significant positive one (p <0.001). There was also a significant positive correlation between WtHR and BSA. On the other hand, BSI was found to be negatively correlated with BMI (r=0.51). Grip strength performance did not significantly correlate with BSI (r=-0.09), though it correlated positively with BMI (r=0.23). Grip strength performance also showed strong positive correlations with peak VO₂ and BSA. 

**Table 2:** Relationship between the indices of body composition, grip strength, and peak VO₂ S - Significant NS - Not significant BMI - Body Mass Index BSA - Body Surface Area BSI - Body Size Index WtHR - Waist to Height ratio

Parameters	Correlation coefficient (r)	P value
BSI vs BMI	-0.51	<0.0001 (S)
BSA vs BMI	0.71	<0.0001 (S)
WtHR vs BMI	0.72	<0.0001 (S)
Peak VO₂ vs BMI	-0.24	0.0425 (S)
Grip strength vs BMI	0.23	0.0746 (NS)
BSA vs BSI	-0.46	0.0002 (S)
WtHR vs BSI	0.18	0.1651 (NS)
Peak VO₂ vs BSI	0.09	0.4903 (NS)
Grip strength vs BSI	-0.09	0.4903 (NS)
WtHR vs BSA	0.30	0.0188 (S)
Peak VO₂ vs BSA	0.02	0.8784 (NS)
Grip strength vs BSA	0.58	<0.0001 (S)
Peak VO₂ vs WtHR	-0.26	0.043 (S)
Grip Strength vs WtHR	0.06	0.646 (NS)
Grip Strength vs Peak VO₂	0.37	0.0033 (S)

**Figure 1:**
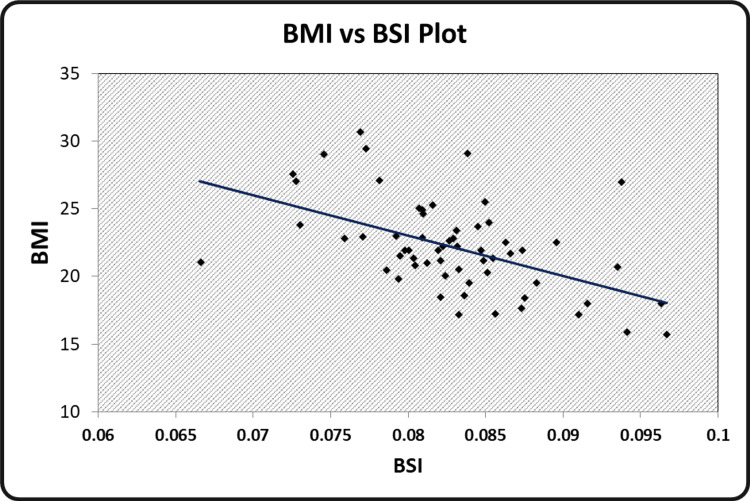
Scatter diagram showing a correlation between BMI and BSI BMI - Body Mass Index BSI - Body Size Index

**Figure 2:**
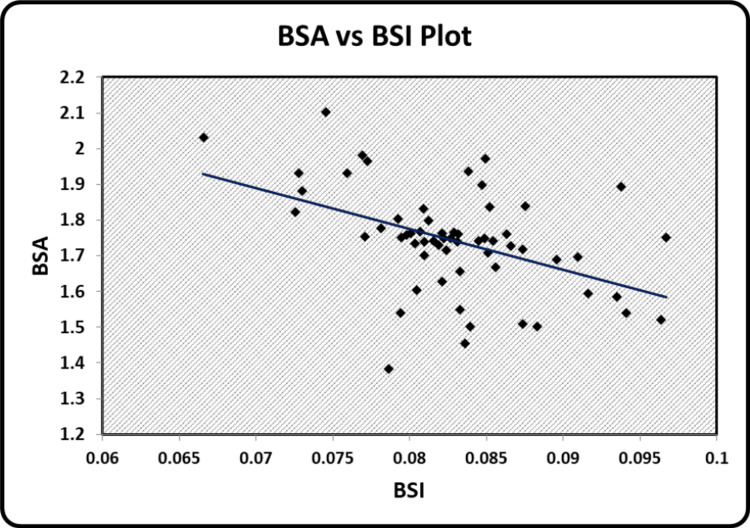
Scatter diagram showing a correlation between BSA and BSI BSA - Body Surface Area BSI - Body Size Index

**Figure 3:**
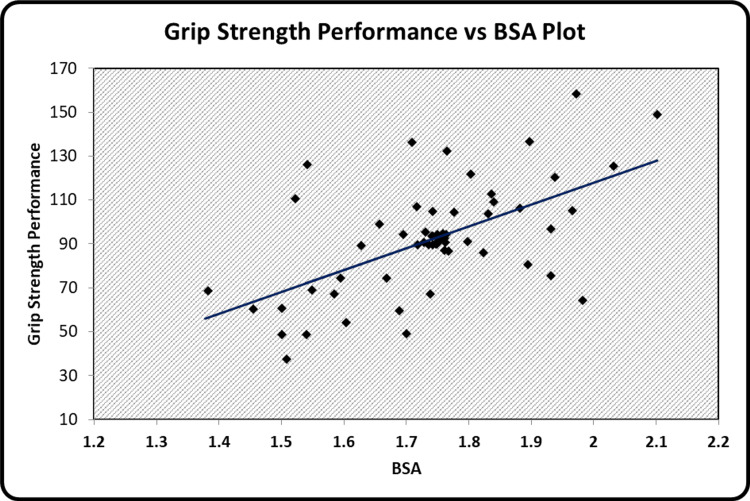
Scatter diagram showing a correlation between grip strength performance and BSA BSA - Body Surface Area

**Figure 4:**
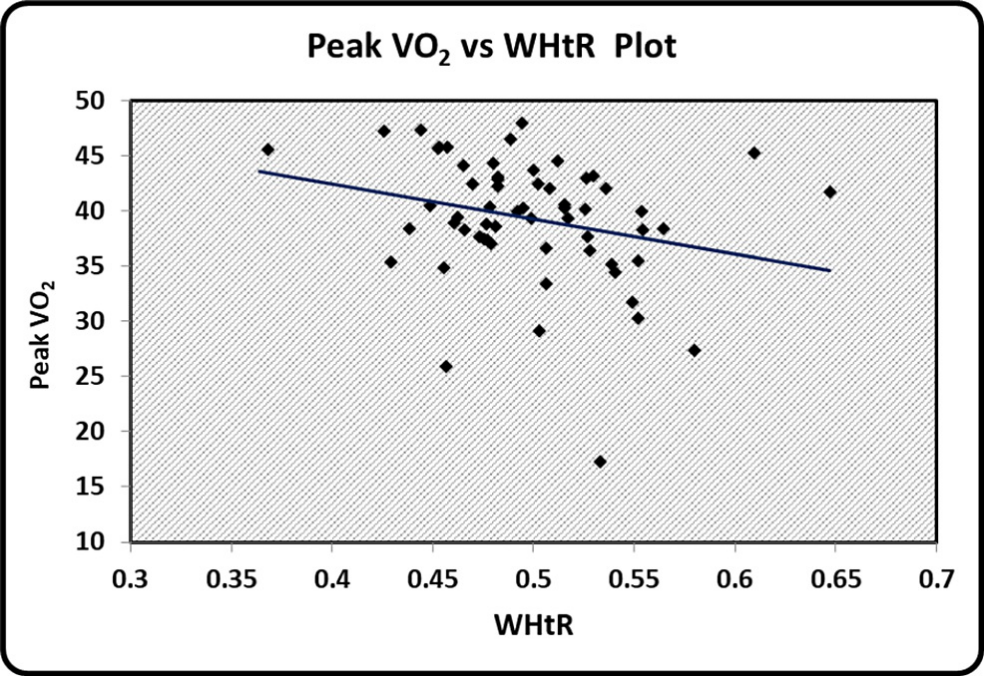
Scatter diagram showing a correlation between peak VO2 and WHtR WtHR - Waist to Height ratio

**Figure 5:**
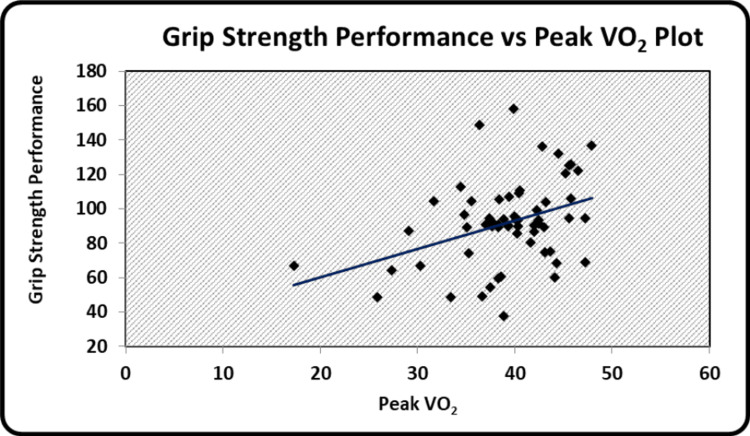
Scatter diagram showing a correlation between grip strength performance and peak VO2

The graphical representation of the significant relationships between the indices of body composition, hand grip strength, and peak VO_2 _has been illustrated. Scatter diagrams showing a correlation between BMI and BSI, BSA and BSI, grip strength performance and BSA, peak VO_2_ and WHtR, grip strength performance and peak VO_2 _can be seen in Figure 1, Figure 2, Figure 3, Figure 4, and Figure 5 respectively. 

## Discussion

Tools and indicators of individual health that are convenient and low cost are the keys to improving the health condition globally, as they allow for better monitoring of conditions that are responsible for large-scale morbidity and mortality. Reduced aerobic fitness and diminished muscular strength with altered body indices resulting in obesity have been associated with premature mortality, but their interactive influence is still unidentified [20]. Therefore, with these aims in mind, this study analyzed the relationship between the indices of body composition with grip strength performance and peak VO₂ to develop an understanding of the interplay between them and to discover the ideal unification of this battery of tests.

Grip strength performance is a leading measure of muscle strength and general health and hence a key parameter in understanding the mortality risks of an individual. When the indices of body composition were compared with the grip strength performance of the subjects, findings suggested that grip strength performance does not significantly correlate with BSI (r=-0.09); however, it correlates positively with BMI (r=0.23). This means subjects with a more central body profile tend to have poorer musculoskeletal health as compared to their peers with the same weight. This understanding is consistent with the observations of Krakauer and Krakauer [1], who concluded that individuals with low grip strength performance along with high BSI have higher mortality, whereas there was no association of BMI with mortality hazards. Therefore, combining BSI with grip strength performance could improve the detection of mortality hazards in a clinical setting.

Peak VO₂ is closely related to the physical fitness of an individual. Studies have shown that, like grip strength performance, peak VO₂ is also a strong and independent risk factor for all-cause mortality, diabetes mellitus, neoplasia, and cardiovascular disease [21]. This importance of measuring aerobic capacity via peak VO_2 _in predicting survival has been reported in asymptomatic populations like those of the Framingham Study [22], the Aerobics Centre Longitudinal Study [23], the Lipid Research Clinics Trial [24], and the Harvard Alumni study [25].

Upon scrutinizing the data acquired from the present study, it was found that peak VO₂ was moderately correlated to grip strength performance (r=0.37) which was statistically significant. This mathematical relationship between peak VO₂ and grip strength performance shows that even though they affect similar health outcomes in individuals, their approach to this indication is not physiologically entangled [20,26].

Furthermore, it was observed that peak VO₂ was independent of BSI and BSA and was found to be weakly correlated to WtHR (r = -0.26) and BMI (r = -0.24). This finding is consistent with that of Carrick-Ranson et al. [27], who found that peak VO₂ values were less in individuals with greater age-related changes when scaled with body surface area but not when represented in absolute values. This establishes the individuality of peak VO₂ and BSI in their indications of overall health and also the need for body composition scaling methods to accurately present the physical effects of aging on health. Since poor physical fitness is a modifiable risk factor, and improvements in fitness over time have been demonstrated to enhance the prognosis, our findings recommend the need for the unison of peak VO₂ with anthropometric parameters for its clinical application [28].

Overall, in the different combinations of relationships between the metrics considered, BSI consistently maintained its individuality and made robust contributions to insights about the comprehensive health of the subjects of our study population. Therefore, based on this exploration of the relationship between the indices of body composition, peak VO₂, and grip strength performance, we recommend BSI based on its supremacy over BMI, from amongst the gamut of anthropometric parameters used, to gain a complete perspective of the health of an individual.

In line with any research endeavor, this study also had a couple of limitations. Aerobic and muscular fitness has been estimated in a cross-sectional manner among central Indian adults due to time constraints as it was short-term research. A longitudinal cohort study is needed to better establish a discrete relationship between aerobic fitness and body composition parameters. Peak VO_2_ testing also has a few drawbacks, like it is a time-consuming test that requires the subject’s enthusiasm. It was quite difficult to get motivated participants for this type of research. Moreover, a different statistical approach could be utilized to evaluate a composite score of multiple indicators, which was not employed in the study but might help the field move forward. 

## Conclusions

The study determined the relationships of grip strength performance and peak VO₂ with the body composition indices and provides a composite health analytical tool to render an overview of the health status of an individual which might mediate the prognosis. Due to its inherent convenience, 'easy to read, easy to analyze' type of results, this non-invasive methodology and rapid output tool can be used in poverty-stricken regions of the world. The outcomes themselves can provide specific health indications exclusive to the realm of the metrics to routinely monitor the overall fitness and the health status of a population.

We recommend the set of BSI, peak VO₂, and grip strength performance, with the scaling of peak VO₂ by BMI or BSA as a comprehensive screening tool. This tool, which embraces peak VO₂ and grip strength performance, could serve for evaluation of the capability of a worker in a physically demanding profession, to set treatment goals, monitor progress during rehabilitation, document the effectiveness of various treatment strategies, and assess the patient’s ability to return to employment.
